# Mapping the neural circuitry of predator fear in the nonhuman primate

**DOI:** 10.1007/s00429-020-02176-6

**Published:** 2020-12-02

**Authors:** Quentin Montardy, William C. Kwan, Inaki C. Mundinano, Dylan M. Fox, Liping Wang, Cornelius T. Gross, James A. Bourne

**Affiliations:** 1 Shenzhen Key Lab of Neuropsychiatric Modulation and Collaborative Innovation Center for Brain Science, Guangdong Provincial Key Laboratory of Brain Connectome and Behavior, CAS Center for Excellence in Brain Science and Intelligence Technology, Brain Cognition and Brain Disease Institute (BCBDI), Shenzhen Institutes of Advanced Technology, Chinese Academy of Sciences, Shenzhen-Hong Kong Institute of Brain Science-Shenzhen Fundamental Research Institutions, Shenzhen, 518055 China; 2 Australian Regenerative Medicine Institute, Monash University, Clayton, VIC 3800 Australia; 3 Epigenetics and Neurobiology Unit, EMBL Rome, European Molecular Biology Laboratory, Via Ramarini 32, 00015 Monterotondo, RM Italy

**Keywords:** Ventromedial hypothalamus, Instinctive predator fear, Connectivity, Mapping, Marmoset, Nonhuman primate

## Abstract

In rodents, innate and learned fear of predators depends on the medial hypothalamic defensive system, a conserved brain network that lies downstream of the amygdala and promotes avoidance via projections to the periaqueductal gray. Whether this network is involved in primate fear remains unknown. To address this, we provoked flight responses to a predator (moving snake) in the marmoset monkey under laboratory conditions. We combined c-Fos immunolabeling and anterograde/retrograde tracing to map the functional connectivity of the ventromedial hypothalamus, a core node in the medial hypothalamic defensive system. Our findings demonstrate that the ventromedial hypothalamus is recruited by predator exposure in primates and that anatomical connectivity of the rodent and primate medial hypothalamic defensive system are highly conserved.

**Electronic supplementary material:**

The online version of this article (10.1007/s00429-020-02176-6) contains supplementary material, which is available to authorized users.

## Introduction

Lesions of the amygdala block the processing of learned and innate fear stimuli in multiple species, including humans (LeDoux 2014; Anderson and Adolphs 2014; Feinstein et al. 2011; Feinstein 2013; Martinez et al. 2011). However, fear induced by internally generated stimuli, such as the inhalation of carbon dioxide, do not require the amygdala (Feinstein et al. 2011). These observations suggest that circuits downstream of the amygdala are sufficient to sustain the behavioral and emotional correlates of fear. This view is supported by extensive work in rodents demonstrating that a circuit from the amygdala to the medial hypothalamus and brainstem, called the medial hypothalamic defensive system, is both necessary and sufficient for innate and learned defensive responses to predators (Canteras 2002; Gross and Canteras 2012; Silva et al. 2013; Markham et al. 2004; Wang et al. 2015; Kunwar 2015). In rodents, sensory inputs are known to project to the medial hypothalamic defensive system via the medial and basomedial nucleus of the amygdala (MeA and BMA) that convey olfactory and polymodal information, respectively (Silva et al. 2016b). the major output of the medial hypothalamic defensive system projects to the dorsal columns of the periaqueductal gray (PAG), via which the system promotes active defensive responses to threat, including freezing and flight (Canteras 2002; Wang et al. 2015; Krieger et al. 1979). Unlike rodents, however, primates typically depend exclusively on visual cues to detect and respond to innate threat cues, yet it remains unknown whether such stimuli are sufficient to recruit the medial hypothalamic defensive system and by what pathway this might occur.

Here we elicited a robust predator-evoked escape response in a nonhuman primate under controlled laboratory conditions, and used c-Fos mapping combined with anatomical tract tracing to identify the neural circuits involved. The common marmoset, *Callithrix jacchus*, is an appealing primate species to study the link between neural circuits and behavior because of its small size and its rich repertoire of affiliative and agonistic social behaviors (Poole et al. 1978; Miller 2016). The defensive responses of marmosets to predators in the wild have been described and include visual scanning, alarm calling, mobbing, avoidance, freezing, and flight (Ferrari and Ferrari 1990; Corrêa and Coutinho 1997). Exposure to toy snakes, cats or raptors has been used in the laboratory setting to induce visual scanning, alarm calling, freezing and threat displays (Vitale et al. 1991; Barros et al. 2000; Barros and Tomaz 2002; Shiba et al. 2015). However, in previously published studies, threat presentation generally necessitated human intervention or handling of the animal and most likely as the result of the gradual presentation of the stimulus, did not elicit robust flight and escape behavior. Our study was designed, on the other hand, to allow sudden and unexpected presentation of a proximal threat and the elicitation of a robust and reliable escape response. To the best of our knowledge, the full repertoire of active defensive responses, including flight and post-flight vigilance, observed in response to predators in the wild has not been reported under laboratory conditions.

## Results

### Snake presentation evokes flight, sustained avoidance and vigilance in the primate

**Fig. 1:**
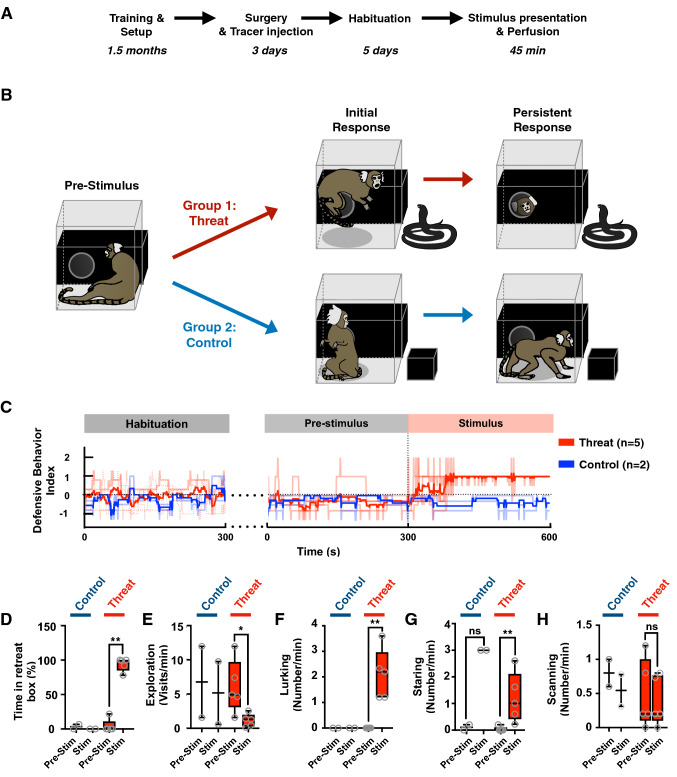
Snake presentation evokes flight, sustained avoidance, and vigilance in the primate. **a** Following an extensive period of habituation training animals underwent surgery for the local delivery of anterograde and retrograde tracers in VMH. After recovery, training was continued for 5 days before exposing the animal to the experimental stimulus. Forty-five minutes after stimulus exposure the animal was anesthetized, perfused, and its brain prepared for cFos immunostaining. **b** Animals were randomly assigned to groups exposed to either an animated rubber snake (threat) or a black cardboard box (control). Images indicate representative behaviors evoked by the stimuli during the initial and persistent response phases of the test. **c** Defensive Behavior Index (light color, individual traces; dark color, mean; 60 s bins) for animals in the threat (red) and control (blue) groups during the final training (habituation), baseline (pre-stimulus) and stimulus exposure (stimulus) sessions; threat exposure during pre-stimulus vs stimulus periods, induced for threat (red) a significant **d** increase in time spent in the retreat box, **e** decrease in exploration, **f** increase in lurking, and **g** increase in staring, but no significant **h** change in scanning (mean of first 5 min; *N* = 5; ****P < 0.0001, **P < 0.01, *P < 0.05)

**Fig. 2:**
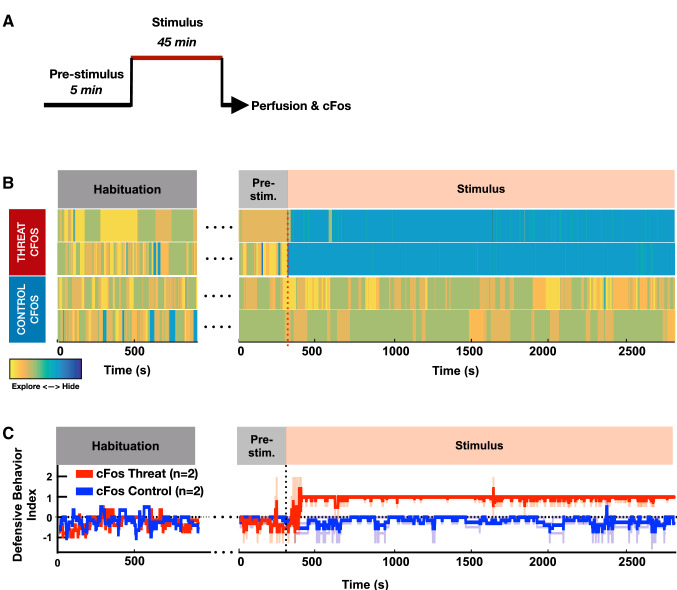
Behavior of animals in the cFos experiment. **a** Experimental phases: 5-min pre-stimulus during which the animal was freely exploring the apparatus, 45-min stimulus when the animal was exposed to the threat (toy animated snake) or control (black box) stimulus by raising a black cloth canopy, and post-experiment processing where the animal was anesthetized and perfused for histology and cFos immunostaining. **b** Defensive Behavior Index, each row corresponding to a different animal in the threat (top) and control (bottom) groups. Color code indicates the animal is hiding in the retreat box (cold) or exploring (warm). **c** Defensive Behavior Index (light color, individual traces; dark color, mean; 60 s bins) for animals in the threat (red) and control (blue) groups during the final training (habituation), baseline (pre-stimulus) and stimulus exposure (stimulus) sessions

Marmosets were pre-trained over 2 months to enter a transparent plexiglass transport box followed by 2–4 weeks of habituation to a testing room, during which time the animal was given access from the transport box to an opaque/dark retreat box via a small door (Fig. 1a, b). Following habituation, the animals underwent MRI-guided stereotaxic surgery (Mundinano et al. 2016) for injection of fluorescent anterograde (dextran amine) and retrograde (cholera toxin B) tracers into the VMH (Fig. 4a, F1982—needle track into left VMH). Following recovery, the animals were trained for an additional 5 days, during which time their baseline behaviors in the isolated testing room were recorded. On the following day, a remotely activated canopy positioned in front of the transport box was raised to reveal the threat stimulus—a moving rubber snake, while control animals were exposed to a neutral stimulus—a small, unmoving black box (Fig. 1b), for a continuous duration of 45 min (Fig. 2a). Exposure to the threat stimulus elicited robust defensive responses in all animals, consisting of jump and flight to the rear of the cage and hypervigilance, and culminating in escape to the adjacent nest box (Fig. 1b, c, Video S1). The behavioral state of the animal was scored using a Defensive Behavior Index, consisting of a weighted sum of distance from the stimulus (from − 0.8 when near the stimulus to + 1 when in the nest box) and defensive behavior (from − 1 when grooming to + 2 during flight; Fig. 1c). As long as the threat stimulus was visible, animals remained principally in the nest box (Fig. 1d, Pre = 4.3% vs Threat = 93%, *P* < 0.001), showing a significant reduction in exploration (Fig. 1e, Pre = 6.0 vs Threat = 1.2, *P* < 0.05) and increase in lurking (Fig. 1f, Pre = 0.0 vs Threat = 2.1, *P* < 0.01) and staring (Fig. 1g, Pre = 0.04 vs Threat = 1.2, *P* < 0.01). Scanning, on the other hand, was not significantly affected by threat presentation (Fig. 1h, Pre = 0.48 vs Threat = 0.39, *P* > 0.5). In comparison with animals exposed to the threat, the Defensive Behavior Index across time of the marmosets exposed to the control stimulus demonstrated no signs of startle or flight (Figs. 1c, 2b, c, Video S2), and mainly remained outside of the nest box with no change in exploration (Fig. 1d–h). During threat presentation animals would occasionally peek out of the nest box and/or briefly enter into the transport box before rapidly retreating back into the nest box (Fig. 2b, top line). To the contrary, in the presence of the control stimulus animals tended to stay closer to the front of the transport box to observe the stimulus (Fig. 1g, Pre = 0.1 vs Stim = 3.0, *P* > 0.5), while scanning behaviors remained unchanged (Fig. 1h). In a subset of animals (*N* = 3) the threat stimulus was presented for 5 min and then hidden again under the canopy to assess whether the latency with which animals would return to the transport box and cease to show defensive behaviors (Fig. S1A). Following removal of the threat stimulus, animals exited the nest box and their Defensive Behavior Index returned to baseline (latency = 43–120 s, Fig. S1B, C). During the post-stimulus period, animals returned to the transport box within 1–2 min, but remained at the rear of the transport box (Fig. S1B). During the post-stimulus period animals spent less time in the nest box than during the stimulus period (Fig. S1D, Threat = 90% vs Recovery = 38%). Other behaviors such as exploration, lurking, and staring also were modulated by the stimulation, although the difference did not reach significance (Fig. S1E–H). A subset of animals were exposed for 45 min to either the threat (*N* = 2) or neutral stimulus (*N* = 2) and processed for histological analysis and cFos expression (Figs. 1a, 2a).

### Threat exposure recruits the primate medial hypothalamic defensive system

**Fig. 3:**
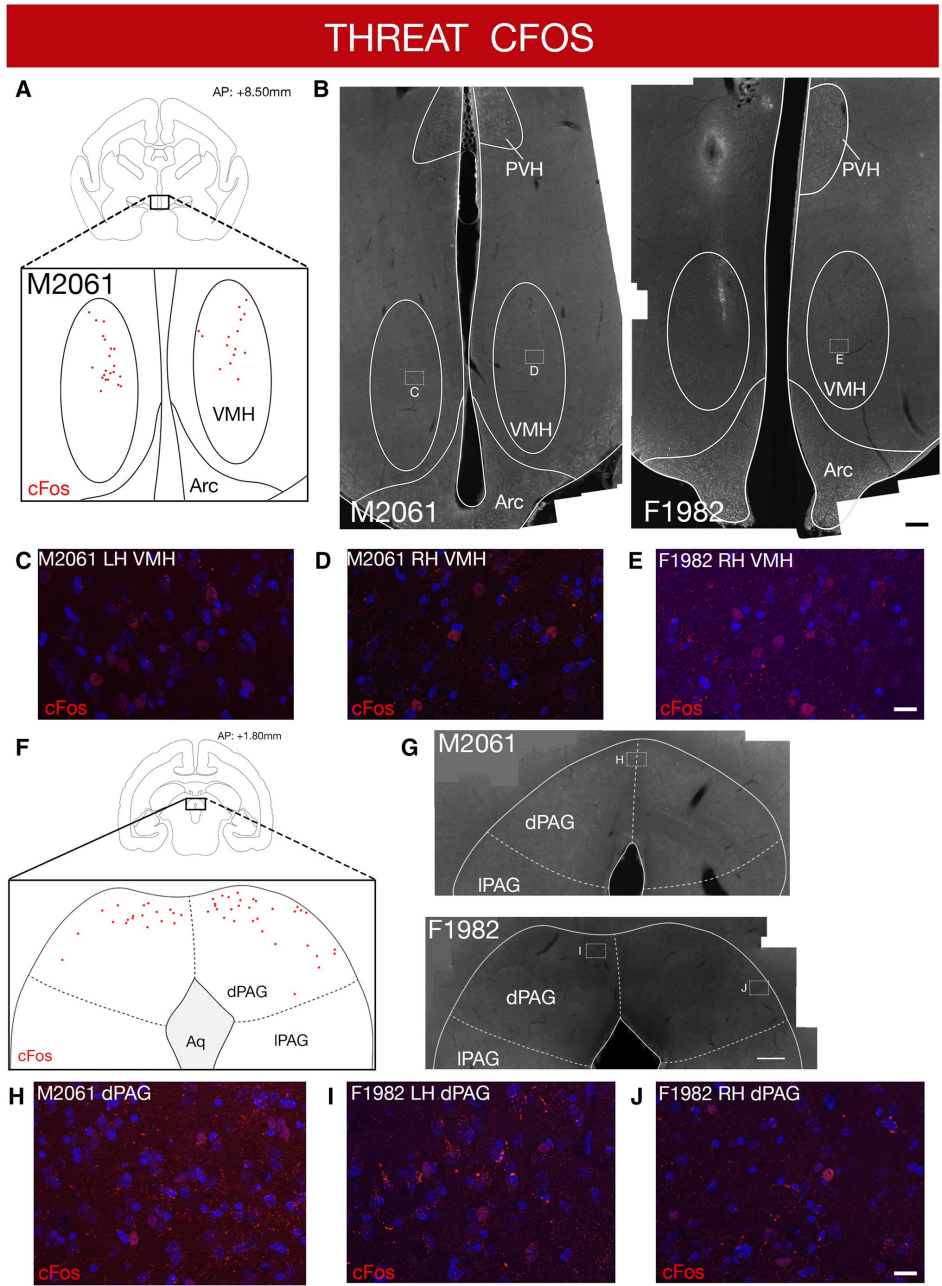
Threat-evoked cFos immunolabel in VMH and PAG. **a** Robust cFos immunostained cells were found in the dorsomedial VMH in animals exposed to snake threat (red dots indicate cFos + cells in representative animal; *N* = 2). **b** Representative coronal sections of the marmoset brain showing cFos immunolabeling in VMH of animals exposed to the threat. **c**–**e** High powered images from insets showing cFos + cells identified within the VMH. The arcuate (Arc) and paraventricular nucleus of the hypothalamus (PVH) contained cFos + cells in both threat and control animals. **f** cFos-immunostained cells were found in the dorsomedial PAG in animals exposed to snake threat. **g** Representative coronal sections of the marmoset brain showing cFos immunolabeling in dPAG of animals exposed to the threat. **h**–**j** High powered images from insets showing cFos + cells identified within the dPAG (numbers indicate animal ID; **a**, **b** scale 200 µm; **c**–**e** scale 20 µm)

To determine whether the medial hypothalamus and its efferent and afferent targets are recruited in primates following predator exposure, we performed cFos immunostaining in brain sections from experimental (Fig. 3) and control (Fig. S2) animals that had previously undergone injection of anterograde and retrograde tracers unilaterally into VMH. Within VMH, cFos + cells were detected in the dorsomedial division (VMHdm), the subnucleus known to be essential for predator defense in rodents and whose activation is sufficient for the induction of fear and panic in humans (Wilent 2010) (Fig. 3a–e). Importantly, no cFos + cells were detected in VMH of control animals (Fig. S2B) or in the ventrolateral division of VMH (VMHvl) known to mediate defensive responses to social threat (Silva et al. 2013; Wang 2019). Consistent with similar studies in rodents (Beijamini and Guimaraes 2006; Baisley et al. 2011) cFos + cells were also detected prominently in experimental, but not control animals in the dorsal periaqueductal grey (dPAG; Fig. 3f–jright, Fig. S2B), a region known to be required for expression of predator defense in rodents (Silva et al. 2016a; Andrade Rufino et al. 2019; Souza and Carobrez 2016). Finally, we identified cFos + cells in the arcuate and paraventricular hypothalamic nuclei (Figs. 3a–e, S2A), although similar numbers of immunopositive cells were seen in experimental and control animals, suggesting generalized recruitment of these structures during behavioral testing. Due to the high immunoreactive background, we were unable to determine the extent of cFos labeling in forebrain areas such as amygdala or medial prefrontal cortex (mPFC).

**Fig. 4:**
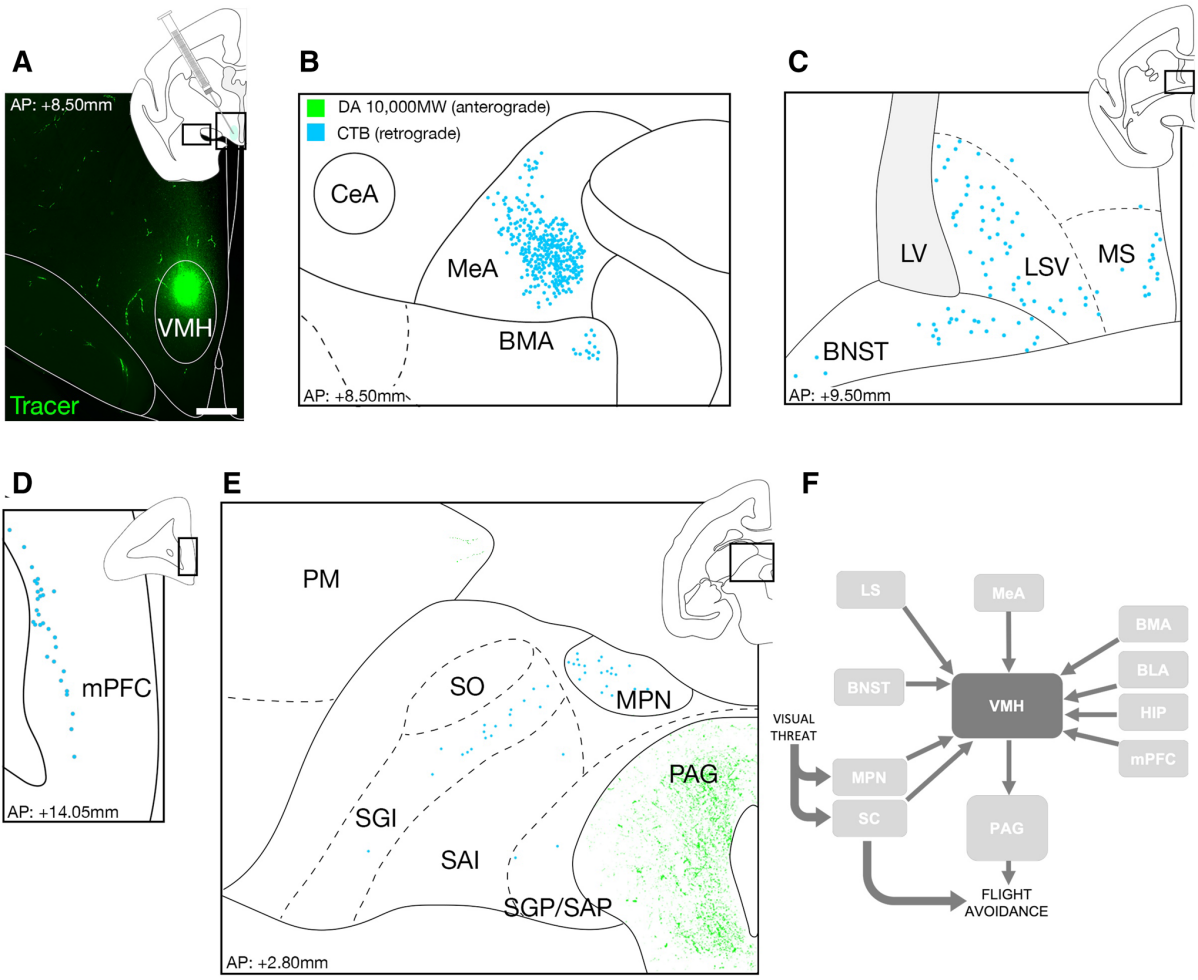
Anterograde and retrograde connectivity of the VMH. **a** Representative coronal section of the marmoset brain showing expression of tracer injected in VMH. **b**–**d** Retrograde and anterograde tracing revealed sparse VMH afferents from mPFC, BNST, LS, MS, BMA and BLA and dense inputs from MeA. **e** Dense VMH efferents were found in PAG and sparse outputs in PM. **f** Summary of the VMH afferents and efferents in the primate. Dark grey arrows denote pathways that were identified in this study

Anterograde and retrograde tracer delivery were restricted to VMH in three out of four animals (Fig. 4a), allowing us to identify afferent and efferent subregions of this nucleus in the primate brain and compare them with similar studies in the rodent. Whilst the phenotype of projecting cells was not investigated with additional morphological analyzes, it is reasonable to interpret them as long-range projection neurons. Sparse retrograde tracer-labeled cell bodies were found in the ventral mPFC (Figs. 4d, S3A, B), medial division of the bed nucleus of the stria terminalis (BNST; Figs. 4c, S3C), ventral division of the medial and lateral septum (respectively MS and LS; Figs. 4c, S3C), posterior basomedial amygdala (BMA; Figs. 4b, S4C, E), and basolateral amygdala (BLA; Fig. S4C, D). Dense retrograde tracer-labeled cell bodies were found in the ventral division of the medial amygdala (MeA; Figs. 4b; S4A, B), consistent with this structure providing significant inputs to VMH in rodents (Canteras et al. 1995). Major anterograde tracer-labeled axonal varicosities, on the other hand, were found in the periaqueductal grey (PAG; Figs. 4e, S5AB, F) and sparse anterograde label was found in the medial pulvinar (PM; Figs. 4e, S5A-C), intermediate layer of the superior colliculus (SGI; Figs. 4e, S5A-B, D) and medial pretectal nucleus (MPN; Figs. 4e, S5AB, E). A few anterograde varicosities were also visible in MeA, BNST, and LS, but these could not be reliably confirmed. Finally, as there is a large proportion of cFos + label colocalized with retrograde tracer label, it is likely that the majority of cFos + label is found in neurons.

## Discussion

It is proposed that visually evoked defensive responses to predators depends on fast, brainstem information processing via retino-collicular projections (Carr 2015; Pessoa and Adolphs 2010; Öhman et al. 2007). From there, threat information passes to mesencephalic motor initiation centers to drive fixed medullary motor programs (Apps 2018; Koutsikou et al. 2017; Dampney 2015). The importance of this pathway is confirmed by the observation that SC lesions in primates abrogate both orienting and anxiety responses to a predator (Maior 2011; DesJardin 2013). At the same time, amygdala lesions also block fear and anxiety responses to predators in primates and humans. However, the mechanism by which the amygdala is recruited and how it might influence defensive behavior remains contested (LeDoux 2007, 2012, 2014; Pessoa and Adolphs 2010). Here we show that the medial hypothalamic defensive system, known to be essential for defensive responses to predator in rodents (Canteras 2002; Gross and Canteras 2012; Silva et al. 2013) is engaged during predator defense in the marmoset.

Critically, our study introduces and validates a new protocol to investigate innate defensive responses to the sudden appearance of a predator-like stimulus in a non-human primate—the marmoset monkey. Previous protocols aimed at eliciting innate defensive responses in non-human primates were primarily focused on measuring the inhibition of approach behavior toward a threat placed in the vicinity of the experimental animal (Barros and Tomaz 2002; Shiba et al. 2015). Here we aimed to elicit robust and reliable flight responses by suddenly revealing an innate threat in close proximity to the marmoset, without obvious human intervention. This feature of our protocol assured the expression of rapid escape behaviors, including flight, in our subjects and represents the first time that explosive flight behaviors has been systematically elicited in primates in a laboratory setting. The animated rubber snake we used as an innate predator-like stimulus evoked robust escape responses. Unfortunately, our use of a static black box as a control object did not allow us to determine whether the effectiveness of our threat stimulus depended on its being animated or rather on shape. Exploring the precise features of our stimuli that drove defensive behaviors would require additional experiments that were beyond the scope of this initial study.

Our findings have two important implications for our understanding of fear in humans. First, our discovery makes it likely that the medial hypothalamus plays a similar role in encoding an internal state of threat in primates as it does in rodents (Kunwar 2015; Silva et al. 2016a; Esteban Masferrer et al. 2018; Krzywkowski et al. 2019; Masferrer et al. 2018; Kennedy et al. 2019). Notably, VMHdm in rodents is required for the induction and expression of both innate and conditioned predator fear (Silva et al. 2013, 2016; Kunwar 2015) and stimulation of VMHdm in monkeys and humans is sufficient to elicit an intense defensive emotional state (Wilent 2010; Lipp and Hunsperger 1978). Furthermore, in humans direct electrical stimulation of the ventromedial hypothalamus (VMH) is sufficient to elicit feelings of intense fear and trigger panic attacks, suggesting that the medial hypothalamus may also participate in human fear (Wilent 2010). The conserved recruitment of medial hypothalamus across species means that these structures must be considered in the search for the etiology and therapeutic treatment of anxiety-related disorders in humans.

Second, our identification of conserved connectivity of the medial hypothalamic defensive system across rodents and primates supports a common function for this system in defensive behaviors. In particular, the discovery that the primate VMH also receives major inputs from MeA, a nucleus known to convey information from the accessory olfactory system in rodents (Swanson and Petrovich 1998; Yao et al. 2017), was unexpected as this sensory system is vestigial in primates (Trotier 2011). These results suggest that MeA may have elaborated its non-olfactory inputs as vision evolved to become the dominant sense in primates. Nevertheless, given that amygdala lesions block fear responses to predators in humans (Feinstein et al. 2011), a parallel, indirect route that brings visual information to forebrain structures and from there to the medial hypothalamus is likely to also be important for both the emotional and behavioral responses to visual threat.

Our tracer data implicate the MeA and BMA in innate defensive behavior in primates—both of which are required for avoidance of predator in rodents (Canteras et al. 1995; Choi 2005; Miller et al. 2019). As in rodents, the major output projections of VMH were found in dPAG, a brainstem structure known to be essential for active response to predator threat, including freezing and flight, and whose function is conserved from rodents (Wang et al. 2015; Krieger et al. 1979; Canteras et al. 1995) to primates (Canteras 2002; Gross and Canteras 2012; Silva et al. 2013; Wang et al. 2015; Kunwar 2015; Mantyh 1982; Markham et al. 2004; Kim 2013; Li and Sheets 2018). Interestingly, the recruitment of dPAG has been found to increase with threat proximity (Mobbs 2007, 2009; Faull and Pattinson 2017; Faul 2020), consistent with the activation of dPAG in our threat protocol. Finally, the existence of inputs from SC and MPN to VMH offers a direct pathway for visual information to rapidly enter the medial hypothalamic defensive system via retino-tectal inputs. Understanding the relative importance of these converging pathways and investigation their conservation in rodents and primates will require the application of loss-of-function approaches (Fig. 4f) (Swanson and Petrovich 1998; Wei 2015; Zhou 2019).

In summary, our data argue for a conserved role of medial hypothalamic instinctive behavior networks across mammals, including humans. Further work is required to assess precisely which aspects of defensive behavior they control and whether they are necessary for generating the conscious emotional states that accompany threat in humans.

## Materials and methods

### Animals

Six Common Marmosets (*C. jacchus*) aged 18–24 months were sourced from the Australian National Nonhuman Primate Breeding and Research Facility. All experiments were conducted in accordance with the Australian Code of Practice for the Care and Use of Animals for Scientific Purposes and were approved by the Monash University Animal Ethics Committee, which also monitored the welfare of these animals.

### Behavioral assay

Testing was conducted in a transparent plexiglass transport box (305 × 295 × 205 mm) connected by a circular opening to a detachable black plexiglass nest box (600 × 140 × 130 mm) and placed on a table in the testing room in front of a removable black cloth canopy that covered the threat and control stimuli, respectively, a black striped rubber toy snake and a square black cardboard box. The snake could be animated by manipulating a pulley system from outside the isolated experimental room. On the day of the stimulation, a separate pulley system was used to lift the canopy, rapidly revealing the stimulus without the need for the experimenter to enter the room. A camera (GoPro Hero4) was positioned in front of the transport box to record the animal’s behavior. Manipulation of the stimulus and monitoring of the animal were all conducted in a room separate from the animal’s experimental room. The experimenter remained out of sight of the animals in a different room during testing. All animals underwent initial training for habituation to the experimental room. During this period, the animals were trained to enter the transport box that had been mounted to their home cage. Once the animals were habituated to the transport box, they were transported daily to the experimental room for habituation. There, the transport box was connected to the nest box, and the animals were allowed to explore the apparatus freely. The duration of habituation sessions was gradually increased from a few minutes to 20 min until the animal remained calm and relaxed during the entire session. At the end of each training session, a reward was given according to the animal’s preference before being brought back to their home cage. All training sessions commenced at 15:00 h. Total time of transport box training and experimental room habituation was approximately 1.5 months. Once all animals were similarly habituated, 4 animals underwent tracer injection surgery and, following 7 days of recovery, an additional 5 days of experimental room habituation to reinforce the training (20 min per session). Following retraining, the animals were exposed to either the control (*N* = 2) or threat stimulus (*N* = 2). Optimization of the control and threat stimulus presentation was conducted with the animal that did not undergo tracer surgery. Experimental validation involved investigating behavioral responses to the threat and control stimuli and assessing post-stimulation (*N* = 3 females, 5 min each: free apparatus exploration, stimulus exposure, stimulus occlusion, free apparatus exploration). For the test experiment, animals were randomly assigned to threat (*N* = 2, one male, one female) or control (*N* = 2, one naive male, and one female previously exposed to the experimental validation) stimulus conditions (5-min free exploration, 45-min stimulus exposure). Following testing, the animals (*n* = 4) were rapidly processed for histological analysis. Animals only undergoing validation protocol (*n* = 2) were not included in histological analyses.

### Behavior assessment

Videos were scored offline using BORIS software (Friard and Gamba 2016). A weighted Defensive Behavior Index was calculated by scoring animals’ location and behaviors, and was represented as a continuously varying measure or heatmap. Animals outside of the nest box were scored 0, and − 1 if inside of the box. Then experimental space, including the transport box, was divided into six zones: close to the threat and crouching/standing (+ 0.5/+ 0.8, respectively), far from the threat whilst crouching/standing (+ 0/+ 0.3), or inside the box whilst peeking (body in the retreat box, head out; + 0.3) or lurking (body and head in the retreat box while keeping the threat in sight; + 0.5). Lurking was considered as both peeking (body in the retreat box, head out) and body and head in the retreat box while keeping the threat in sight. Flight (− 2), freezing and scanning (− 0.5) and grooming behaviors (+ 1) were also computed. Exploration was estimated by counting the passage of the animal between zones. For several measures, data from both groups of animals were included as the protocols were indistinguishable over the first 10 min of testing. Staring was scored by counting each time the animal looked at the stimulation area. Scanning was scored by counting each time the animal looked upwards and around. Due to a limited sample number, all behaviors were statistically analyzed by Mann–Whitney *U* test. Statistical analyses were performed using Graphpad Prism 8.4.

### Surgery

Preparation of the animals for MRI-guided microinjection of the VMH was performed as previously described (Mundinano et al. 2016). In brief, animals were anaesthetized and scanned in a 9.4 T small-bore animal scanner. To facilitate reconstruction of the marmoset brain and visualization of the VMH structural T2 images were acquired with parameters included the following—repetition time/echo time: 6000/40 ms, echo train length: 4, field of view: 38.4 × 38.4 mm^2^, acquisition matrix: 192 × 192, 100 coronal slices adjusted according to the size of the brain, slice thickness: 0.4 mm, signal averages: 4, scan time: 19 min, 42 s. Subsequently, the left hemisphere VMH was pressure injected with 180 nl of a bi-directional neural tracer cocktail consisting of 5 µg/µl retrograde Cholera toxin subunit B conjugated with Alexa Fluor 488 (Life Technologies, cat #C22841) and 50 µg/µl anterograde dextran amine 10,000 MW conjugated with Alexa Fluor 488 (Life Technologies, cat #D22910). Animals were allowed 1 week to recover to facilitate transport of neural tracers. Following 7 days of recovery, animals underwent behavioral testing.

### Tissue processing

At the conclusion of behavioral testing, animals were deeply anaesthetized with 100 mg/kg sodium pentobarbitone. Following apnea, animals were transcardially perfused with heparinized saline and 4% paraformaldehyde in 0.01 M PBS. Brains were post-fixed for 24 h in 4% PFA before being serially dehydrated in sucrose (10%, 20%, and 30%) solutions before being snap-frozen in – 50 °C isopentane and cryosectioned in the coronal plane at 50 µm. Sections were divided into four series and stored free-floating in a cryoprotective solution consisting of 50% phosphate buffered saline (PBS), 30% ethylene glycol and 20% glycerol at – 20 °C. For each subject, a full series was mounted onto glass slides (Superfrost plus), dehydrated in serial alcohols and cleared in xylene before being mounted in DPX for analysis of tracer label.

### Histology and immunohistochemistry

Tissue sections were rinsed in PBS before undergoing pre-treatment in a blocking solution consisting of PBS with 10% normal donkey serum and 2% Triton-X for 1 h at room temperature. Following blocking sections were incubated with primary antibody in pre-treatment solution for 16–18 h at 4 °C. Sections were then washed in PBS before incubation in donkey anti-rabbit Alexa Fluor 594 secondary antibody (1:1000, Life Technologies, cat #A11058) for 1 h at room temperature and washed and counterstained with Hoechst (Life Technologies, cat #H3569). Primary antibodies used in this study were rabbit anti-cFos (1:1000, Sigma Aldrich, cat #F7799) to assess neural activity and rabbit anti-parvalbumin (1:2000, Swant cat #PV27) to delineate boundaries of the superior colliculus. Acetylcholinesterase staining was performed to allow for demarcation of amygdala subnuclei and layers of the superior colliculus. The staining protocol was adapted from previous studies (Paul et al. 2010; Hardy et al. 1976).

### Microscopy and image processing

Imaging was performed on an Axio Imager Z1 microscope (Zeiss). Images were acquired with a Zeiss Axiocam HRm digital camera using Axiovision software (v. 4.8.1.0). The objectives used were Zeiss EC‐Plan Neofluar 5 × 0.16, #420330‐9901, EC‐Plan Neofluar 10 × 0.3, #420340‐9901; Plan Apochromat 20 × 0.8 #420650‐9901; EC Plan Neofluar 40 × 1.3 oil #420462‐9900. Filter sets used were Zeiss DAPI #488049-9901-000, Zeiss HE eGFP #489038‐9901‐000 and Zeiss HQ TR #000000-1114-462. Stitching of images and adjustments to contrast and brightness were performed using Adobe Photoshop CC2015. The line art, boundaries and contours for all figures were executed using Adobe Illustrator CC2015. Demarcation of areas was achieved with AChE and parvalbumin labeling and compared with the Marmoset Brain in Stereotaxic Coordinates (Paxinos et al. 2012).

## Electronic supplementary material

Below is the link to the electronic supplementary material.

Supplementary file1 (MP4 34364 KB)

Supplementary file2 (MP4 34358 KB)

Supplementary file3 Figure S1. Behavior of animals in the post-stimulation experiment. (A) Experimental phases: 5 min pre-stimulus during which the animal was freely exploring the apparatus, 5 min stimulus when the animal was exposed to the threat (toy animated snake) stimulus by raising a black cloth canopy, and 5 minutes post-stimulation period when the stimulus was covered again by the cloth canopy. (B) Defensive behavior index, each row corresponding to a different animal. Color code indicates the animal is hiding in the retreat box (cold) or exploring (warm). (C) Defensive Behavior Index (light color, individual traces; dark color, mean; 60 sec bins) for animal during the habituation, pre-stimulus, threat, and post-stimulation phases. (D-H) Quantification of behaviors during the pre-threat, threat, and post-stimulation phases (N = 3; **P < 0.01, *P < 0.05). Figure S2. Control-evoked cFos immunolabel in VMH and PAG. (A) Representative coronal sections of the marmoset brain for animals exposed to the control stimulus, showing no cFos+ cells were identified in VMH . The arcuate (Arc) and paraventricular nucleus of the hypothalamus (PVH) contained cFos+ cells in control animals. (B) No cFos immunolabeling could be found in dPAG of animals exposed to the control stimulation (numbers indicate animal ID; A, B: scale = 200 µm; C-E: scale = 20µm). Figure S3. VMH connectivity in the forebrain. Representative coronal sections of the marmoset brain into which retrograde and anterograde tracers were delivered into VMH showing retrograde labeled cell bodies in (A, B high resolution inset) medial prefrontal cortex (mPFC) and (C) bed nucleus of the stria terminalis (BNST) and lateral and medial septum (LS and MS, respectively; scale: A 500 µm, B 100 µm, C 200 µm). Figure S4. VMH connectivity in amygdala. Representative coronal sections of the marmoset brain into which retrograde and anterograde tracers were delivered into VMH showing retrograde labeled cell bodies in (A, high resolution insets B-C) BLA and BMA and (D, high resolution inset E) MeA (grayscale inset shows acetylcholinesterase stained adjacent section that was used to delineate brain regions; scale: A 1000 µm, B-C and E 50 µm, D 100µm). Figure S5. VMH connectivity in midbrain and thalamus. Representative coronal sections of the marmoset brain into which retrograde and anterograde tracers were delivered into VMH showing anterograde labeled processes in (A-B low resolution images with insets C-F; AChE and Parvalbumin to delineate anatomical boundaries) PAG and PM, and retrograde labeled cell bodies in the SGI and MPN (scale: C 50 μm, D-E 20 μm and F 100μm). (PDF 14177 KB)
