# Supplementary figures and images for: Mapping the neural circuitry of predator fear in the nonhuman primate
Source: Brain Struct Funct. 2020 Dec 2;226(1):195–205. doi: 10.1007/s00429-020-02176-6 (PMC7817595; doi:10.1007/s00429-020-02176-6)

**Fig. S1**

**A**

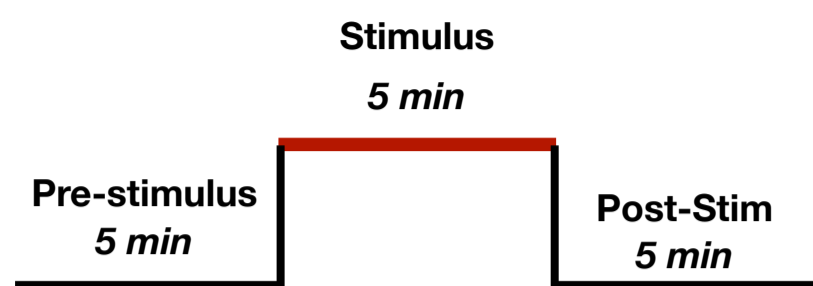

**B**

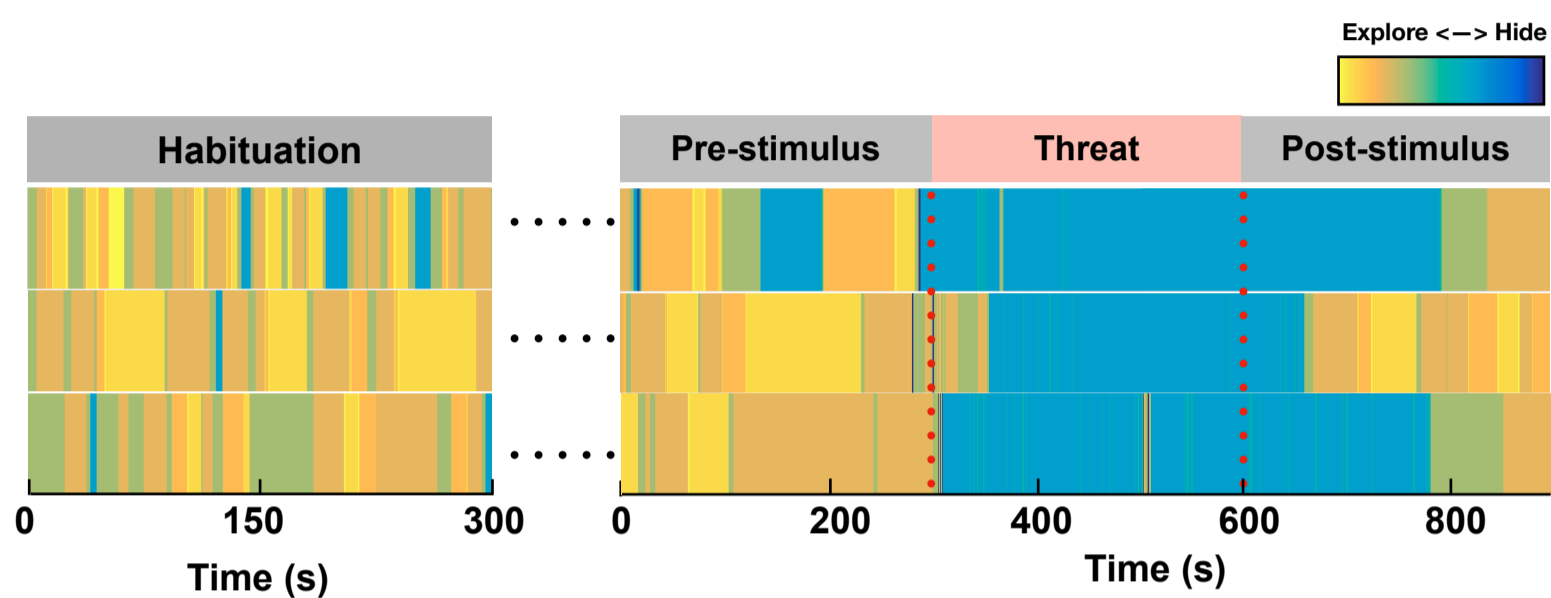

**C**

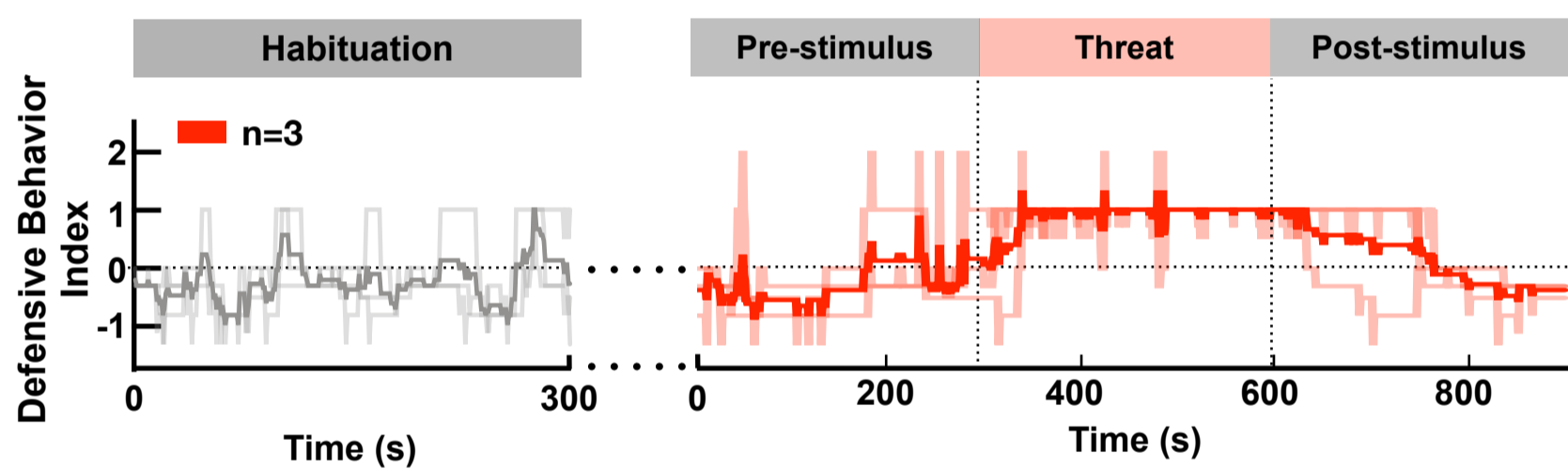

**D**

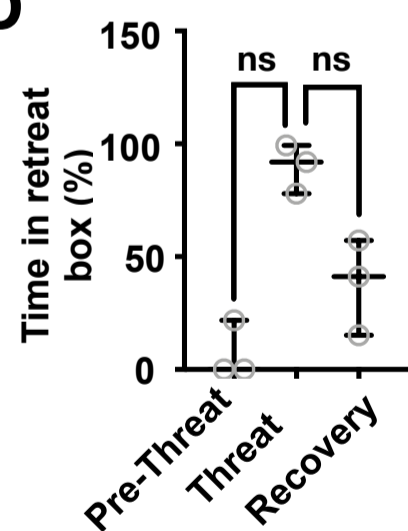

**E**

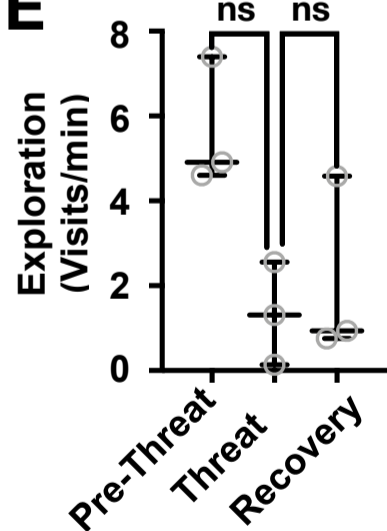

**F**

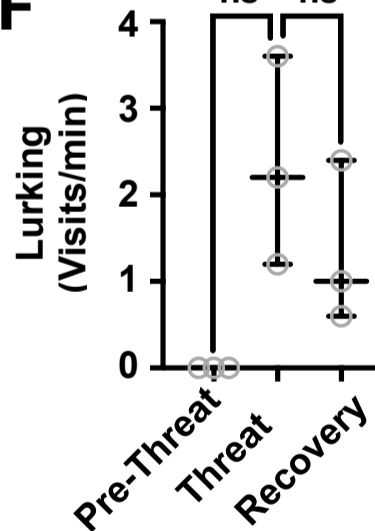

**G**

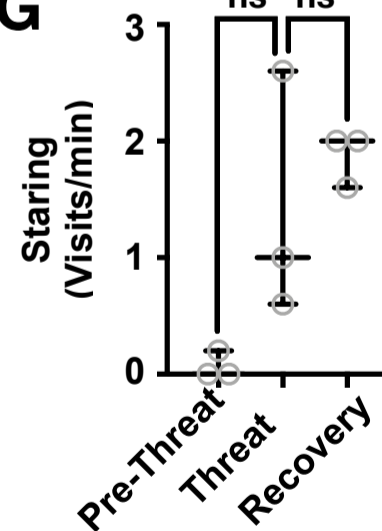

**H**

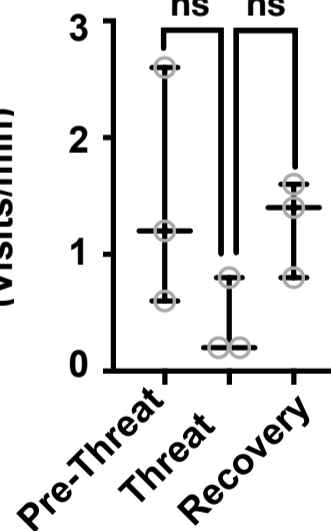

Fig. S2

CONTROL CFOS

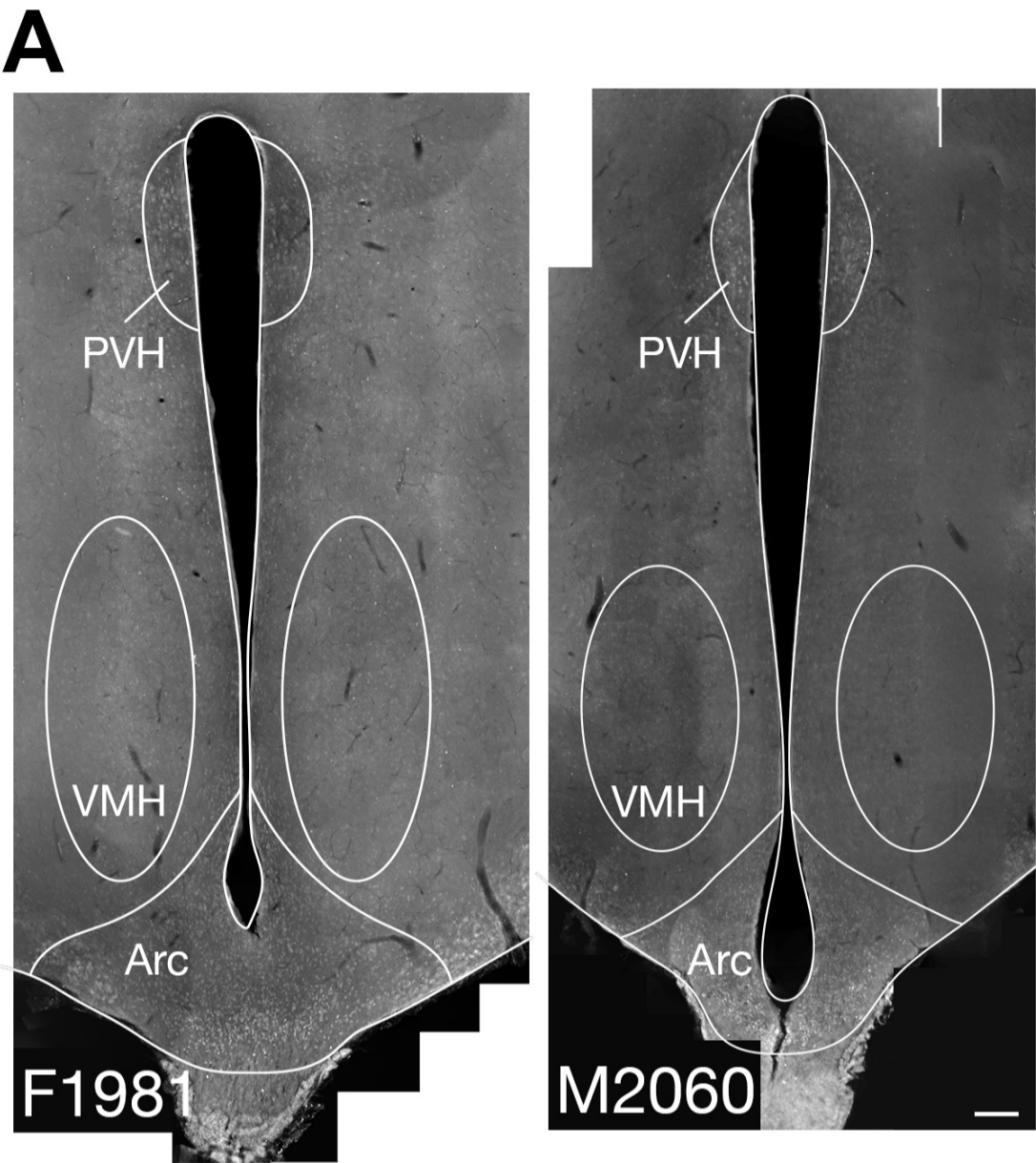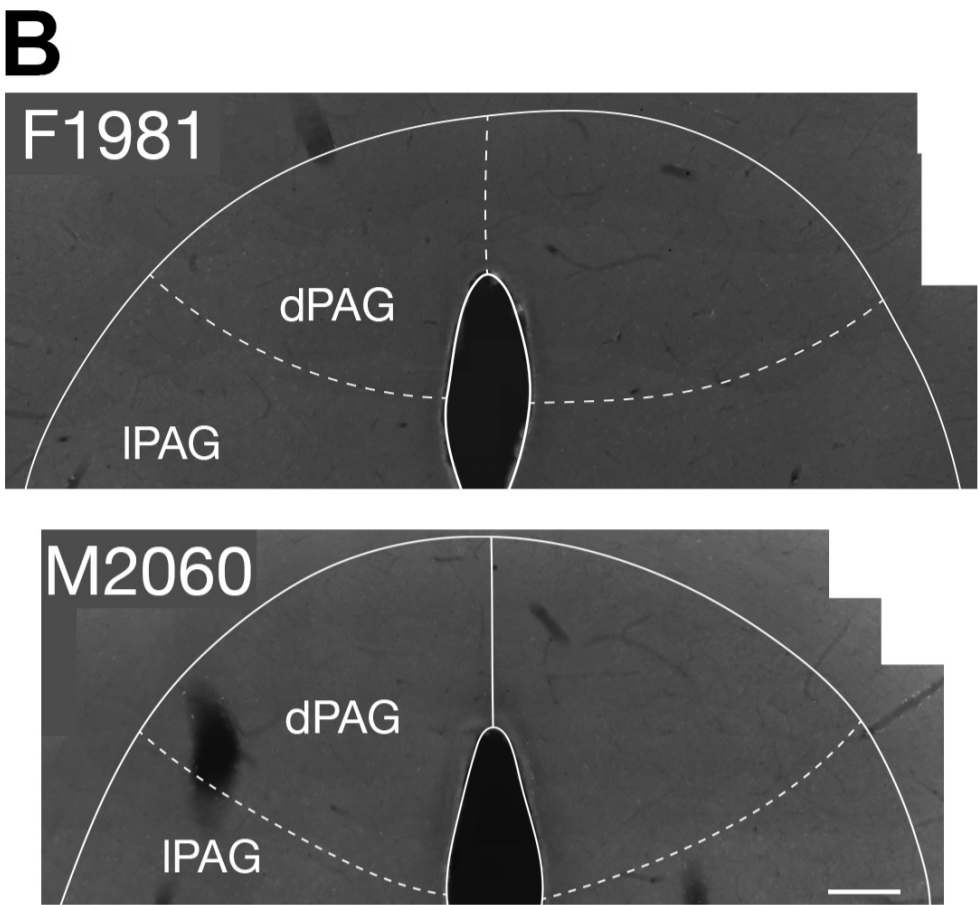

**Fig. S3**

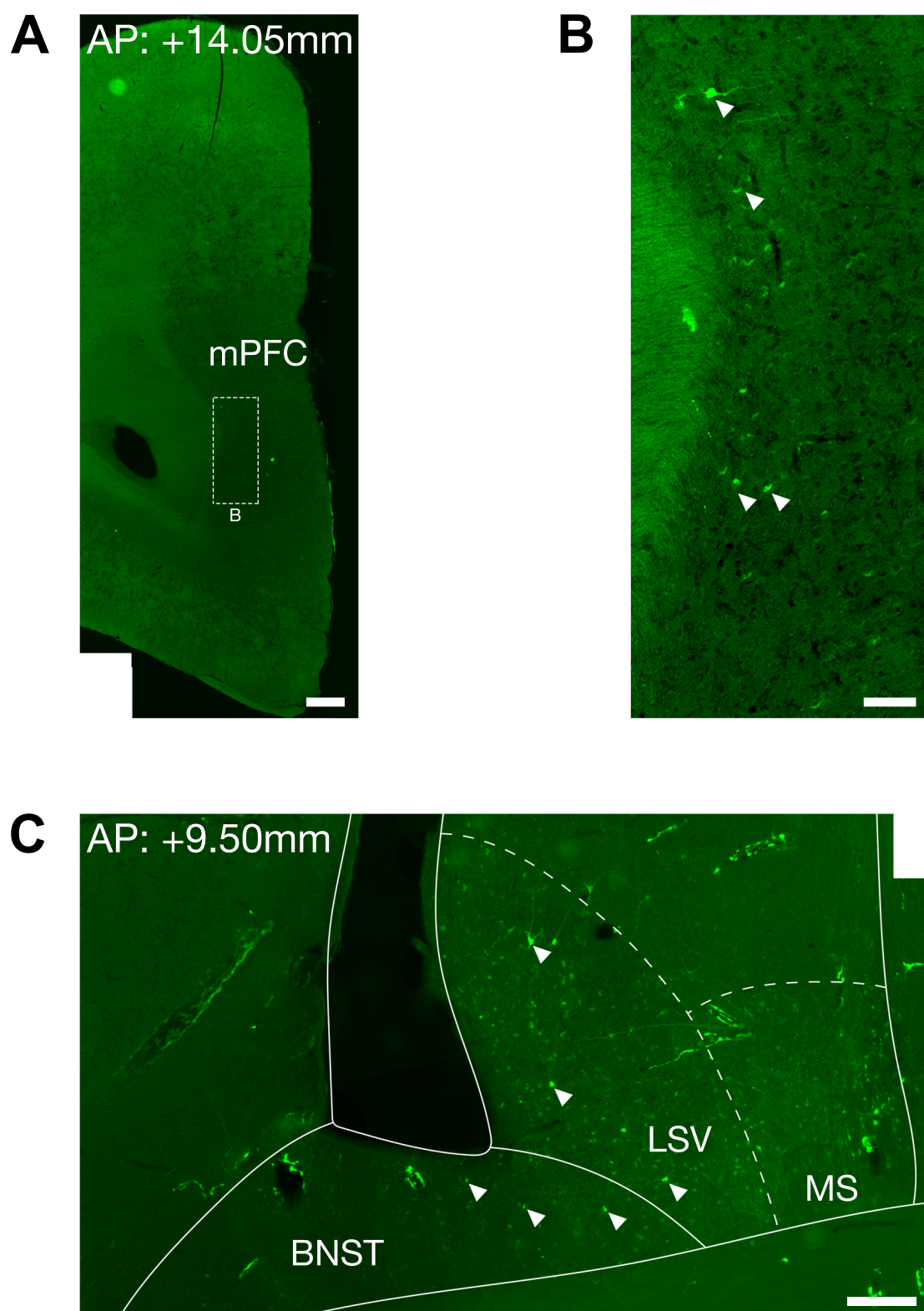

**Fig. S4**

**A** AP: +9.15mm

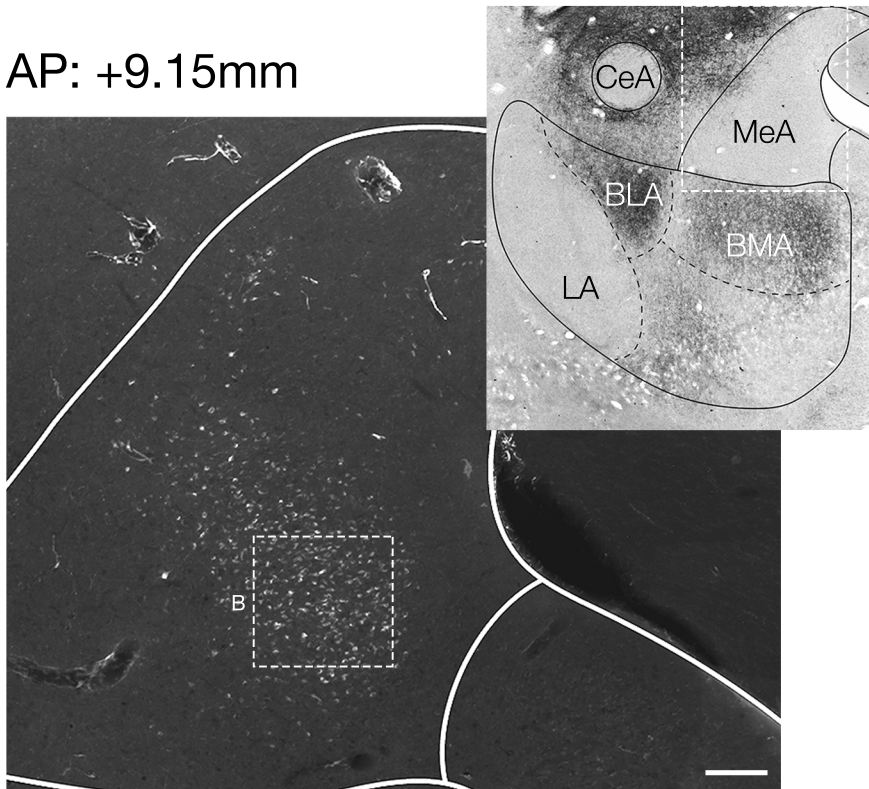

**B**

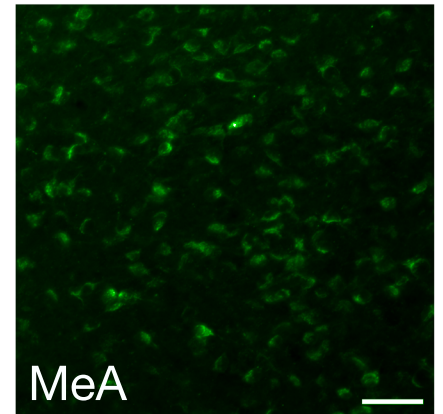

**C** AP: +8.65mm

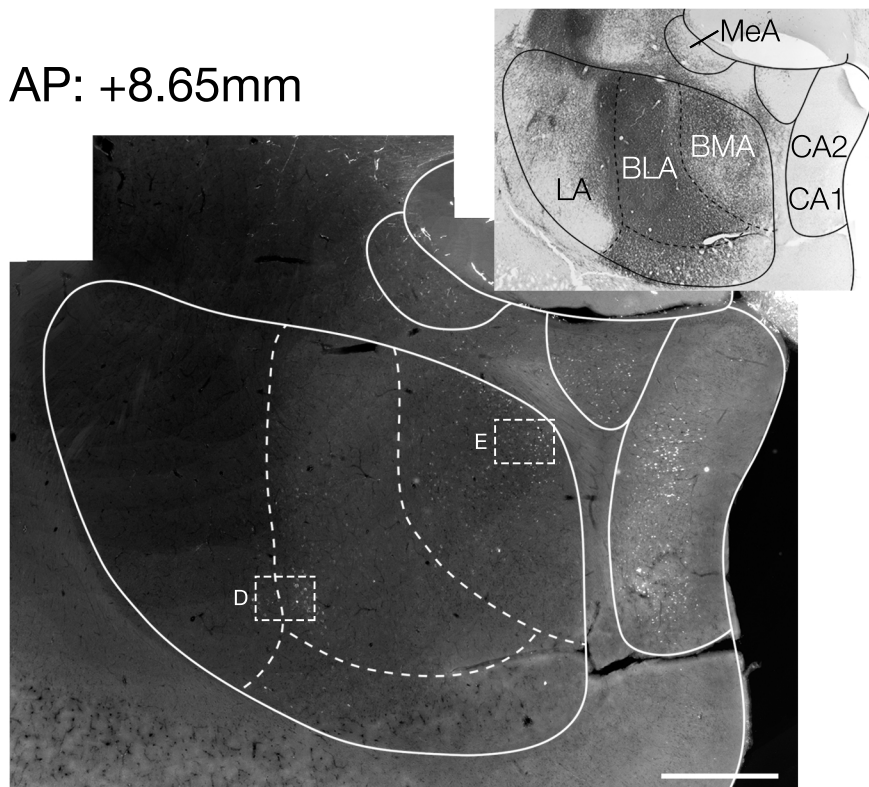

**D**

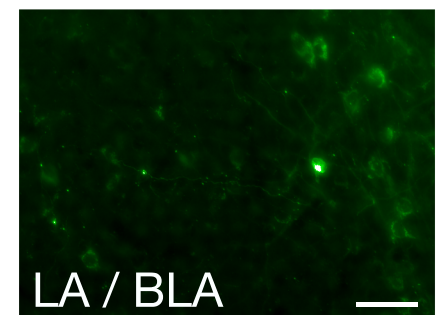

**E**

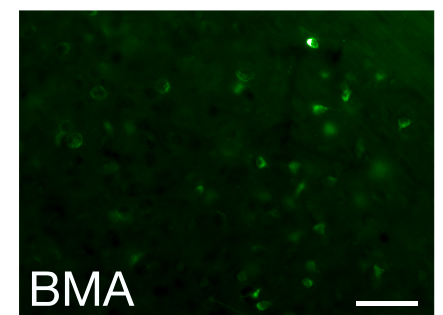

**Fig. S5**

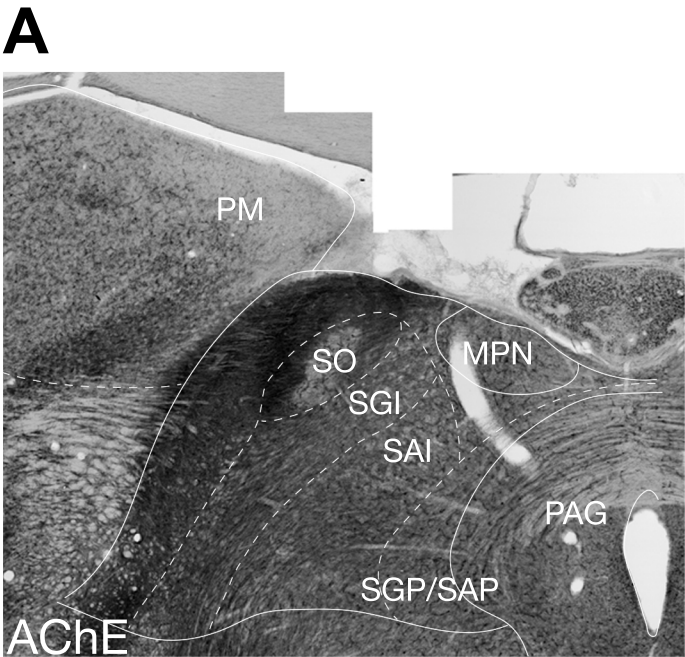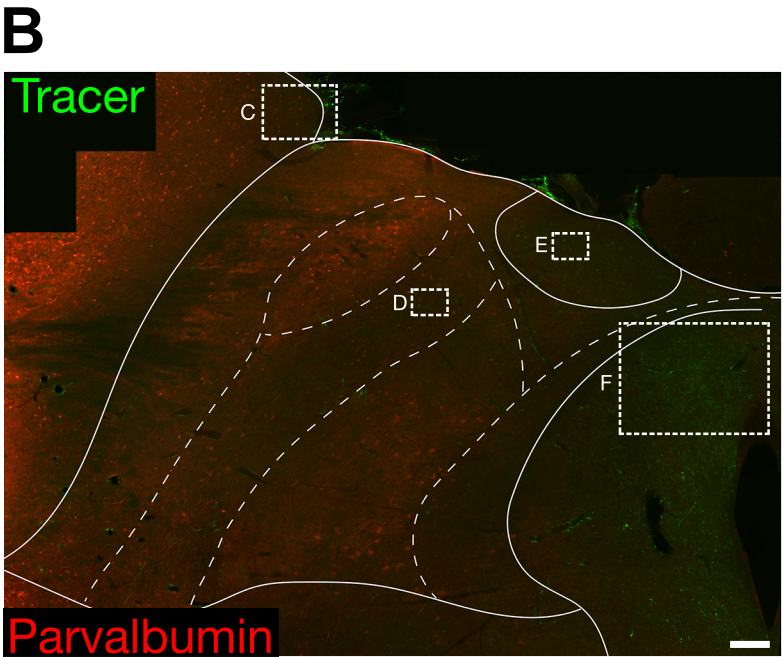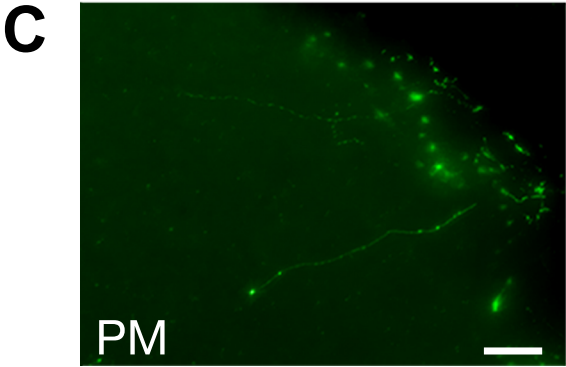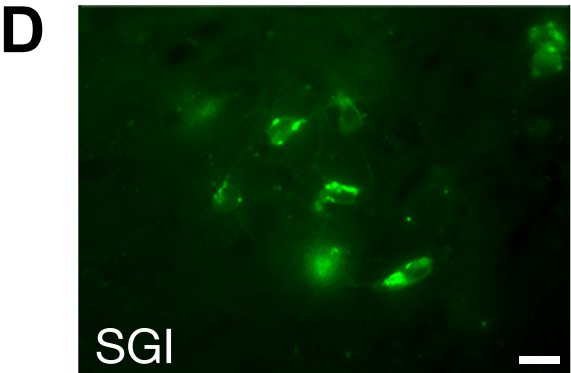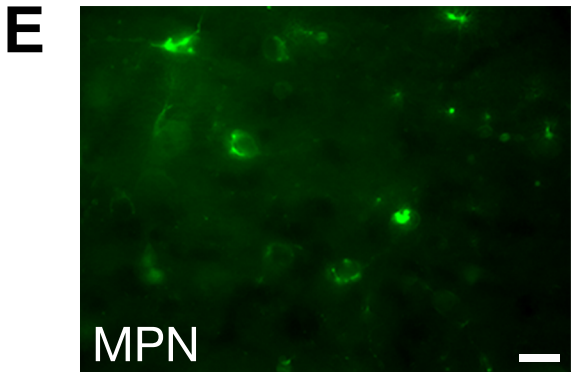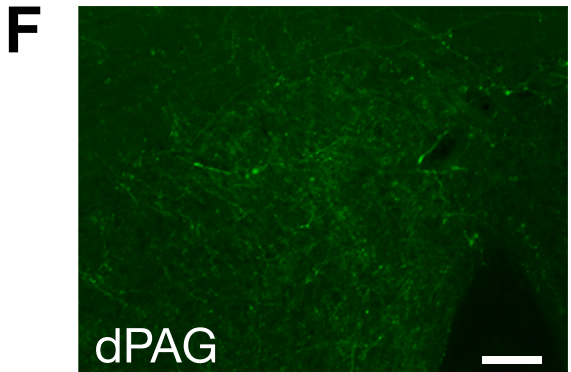

Supplement: Supplementary file 3 — Supplementary file3 Figure S1. Behavior of animals in the post-stimulation experiment. (A) Experimental phases: 5 min pre-stimulus during which the animal was freely exploring the apparatus, 5 min stimulus when the animal was exposed to the threat (toy animated snake) stimulus by raising a black cloth canopy, and 5 minutes post-stimulation period when the stimulus was covered again by the cloth canopy. (B) Defensive behavior index, each row corresponding to a different animal. Color code indicates the animal is hiding in the retreat box (cold) or exploring (warm). (C) Defensive Behavior Index (light color, individual traces; dark color, mean; 60 sec bins) for animal during the habituation, pre-stimulus, threat, and post-stimulation phases. (D-H) Quantification of behaviors during the pre-threat, threat, and post-stimulation phases (N = 3; **P < 0.01, *P < 0.05). Figure S2. Control-evoked cFos immunolabel in VMH and PAG. (A) Representative coronal sections of the marmoset brain for animals exposed to the control stimulus, showing no cFos+ cells were identified in VMH . The arcuate (Arc) and paraventricular nucleus of the hypothalamus (PVH) contained cFos+ cells in control animals. (B) No cFos immunolabeling could be found in dPAG of animals exposed to the control stimulation (numbers indicate animal ID; A, B: scale = 200 µm; C-E: scale = 20µm). Figure S3. VMH connectivity in the forebrain. Representative coronal sections of the marmoset brain into which retrograde and anterograde tracers were delivered into VMH showing retrograde labeled cell bodies in (A, B high resolution inset) medial prefrontal cortex (mPFC) and (C) bed nucleus of the stria terminalis (BNST) and lateral and medial septum (LS and MS, respectively; scale: A 500 µm, B 100 µm, C 200 µm). Figure S4. VMH connectivity in amygdala. Representative coronal sections of the marmoset brain into which retrograde and anterograde tracers were delivered into VMH showing retrograde labeled cell bodies in (A, high [file 429_2020_2176_MOESM3_ESM.pdf]
